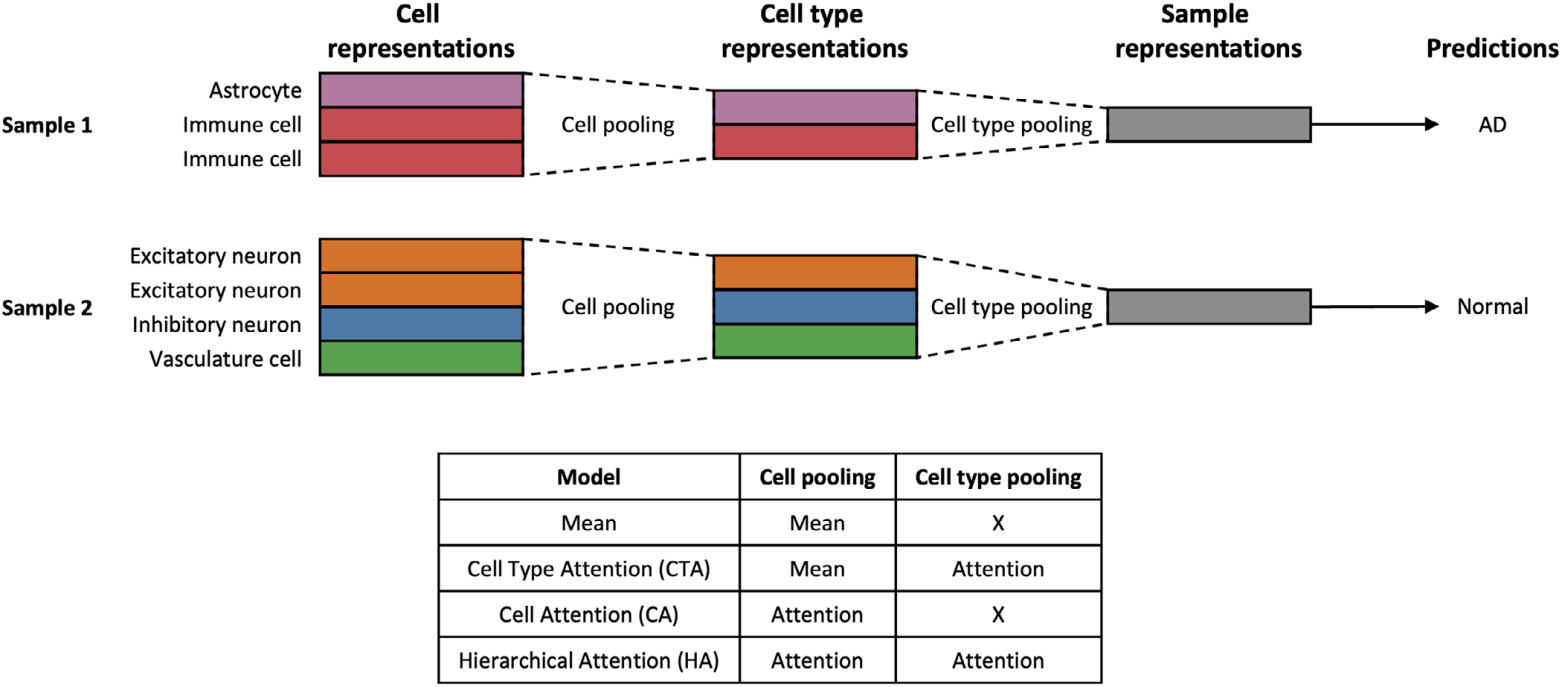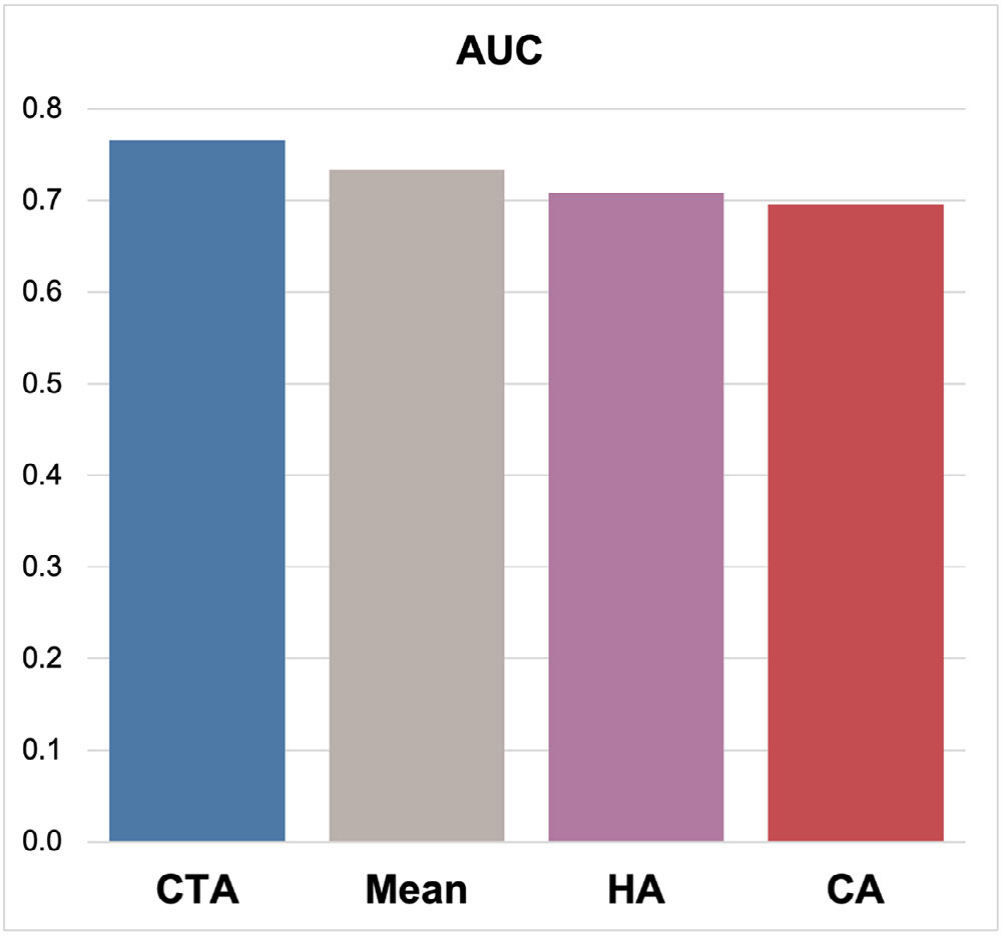# Cell Type‐Aware Multiple Instance Learning Improves Alzheimer’s Disease Prediction from snRNA‐seq

**DOI:** 10.1002/alz70861_108521

**Published:** 2025-12-23

**Authors:** Soorin Yim, Kyungwook Lee, Dongyun Kim, Sungjoon Park, Doyeong Hwang, Kiyoung Kim, Amy R Dunn, Daniel M Gatti, Elissa J Chesler, Kristen MS O'Connell, Soonyoung Lee

**Affiliations:** ^1^ LG AI Research, Gangseo‐gu, Seoul Korea, Republic of (South); ^2^ The Jackson Laboratory, Bar Harbor, ME USA

## Abstract

**Background:**

Alzheimer’s disease (AD) is characterized by complex, cell‐type‐specific molecular changes. Single‐nucleus RNA sequencing (snRNA‐seq) enables detailed analysis of these alterations, offering insights into AD pathogenesis. Predictive models that classify disease status from snRNA‐seq data can aid interpretation by linking expression patterns to patient phenotypes. However, most existing models ignore cell types, limiting both accuracy and biological interpretability.

**Method:**

We applied a recently developed hierarchical multiple instance learning (MIL) framework to improve patient‐level phenotype prediction from single‐nucleus RNA‐Seq (snRNA‐seq) data. This model performs two‐step pooling: first, cell representations are aggregated within each cell type using either mean or attention‐based pooling; then, cell type representations are aggregated into a sample embedding via attention mechanism. We compared this hierarchical approach to models that ignore cell‐type structure, using the ROSMAP cohort, which comprises postmortem snRNA‐seq profiles from individuals with Alzheimer’s disease and cognitively normal controls.

**Result:**

Among four models, Cell Type Attention (CTA) achieved superior predictive performance on the ROSMAP dataset, improving the area under the ROC curve (AUC) for AD vs. control classification. Moreover, CTA offers improved interpretability by enabling attribution of prediction importance at cell‐type levels. Using this framework, we identified key cell types contributing to the classification outcome, highlighting disease‐relevant populations such as astrocytes.

**Conclusion:**

By applying a hierarchical MIL to Alzheimer’s snRNA‐seq data, we demonstrate enhanced predictive performance and interpretability over existing models. This approach not only improves classification of AD status but also facilitates the identification of cell types most associated with disease, offering insights that may support biomarker discovery and therapeutic development.